# Progression of non-alcoholic steatosis to steatohepatitis and fibrosis parallels cumulative accumulation of danger signals that promote inflammation and liver tumors in a high fat–cholesterol–sugar diet model in mice

**DOI:** 10.1186/s12967-015-0552-7

**Published:** 2015-06-16

**Authors:** Michal Ganz, Terence N Bukong, Timea Csak, Banishree Saha, Jin-Kyu Park, Aditya Ambade, Karen Kodys, Gyongyi Szabo

**Affiliations:** Department of Medicine, University of Massachusetts Medical School, 364 Plantation St, LRB-208, Worcester, MA 01605 USA

**Keywords:** Non-alcoholic steatohepatitis (NASH), High fat diet with high cholesterol and a high sugar supplement (HF–HC–HSD), Inflammasome, Danger signals, Tumor

## Abstract

**Background:**

Non-alcoholic fatty liver disease (NAFLD) is becoming a pandemic. While multiple ‘hits’ have been reported to contribute to NAFLD progression to non-alcoholic steatohepatitis (NASH), fibrosis and liver cancer, understanding the natural history of the specific molecular signals leading to hepatocyte damage, inflammation and fibrosis, is hampered by the lack of suitable animal models that reproduce disease progression in humans. The purpose of this study was first, to develop a mouse model that closely mimics progressive NAFLD covering the spectrum of immune, metabolic and histopathologic abnormalities present in human disease; and second, to characterize the temporal relationship between sterile/exogenous danger signals, inflammation, inflammasome activation and NAFLD progression.

**Methods:**

Male C57Bl/6 mice were fed a high fat diet with high cholesterol and a high sugar supplement (HF–HC–HSD) for 8, 27, and 49 weeks and the extent of steatosis, liver inflammation, fibrosis and tumor development were evaluated at each time point.

**Results:**

The HF–HC–HSD resulted in liver steatosis at 8 weeks, progressing to steatohepatitis and early fibrosis at 27 weeks, and steatohepatitis, fibrosis, and tumor development at 49 weeks compared to chow diet. Steatohepatitis was characterized by increased levels of MCP-1, TNFα, IL-1β and increased liver NASH histological score. We found increased serum levels of sterile danger signals, uric acid and HMGB1, as early as 8 weeks, while endotoxin and ATP levels increased only after 49 weeks. Increased levels of these sterile and microbial danger signals paralleled upregulation and activation of the multiprotein complex inflammasome. At 27, 49 weeks of HF–HC–HSD, activation of M1 macrophages and loss of M2 macrophages as well as liver fibrosis were present. Finally, similar to human NASH, liver tumors occurred in 41% of mice in the absence of cirrhosis and livers expressed increased p53 and detectable AFP.

**Conclusions:**

HF–HC–HSD over 49 weeks induces the full spectrum of liver pathophysiologic changes that characterizes the progression of NAFLD in humans. NAFLD progression to NASH, fibrosis and liver tumor follows progressive accumulation of sterile and microbial danger signals, inflammasome activation, altered M1/M2 cell ratios that likely contribute to NASH progression and hepatic tumor formation.

**Electronic supplementary material:**

The online version of this article (doi:10.1186/s12967-015-0552-7) contains supplementary material, which is available to authorized users.

## Background

The prevalence of obesity is increasingly recognized as a pandemic that has been responsible for over 3.4 million deaths in 2010 [1]. This condition affects both adults and children at an alarming rate with the well recognized adverse health risk for the development of metabolic diseases including non-alcoholic fatty liver disease (NAFLD). It has been reported that about 30% of the Western population suffers from NAFLD that increases to about 75% in obese individuals [2, 3]. NAFLD includes a wide spectrum of disease stages, ranging from steatosis alone, which is characterized by at least 5% triglyceride accumulation, to nonalcoholic steatohepatitis (NASH), which is characterized by liver fat accumulation and inflammation. Fibrosis is seen in 20% of NASH patients and hepatocellular carcinoma (HCC) develops in NASH livers even without cirrhosis [4–6]. Current treatment options for NAFLD are limited and ineffective [7, 8].

Non-alcoholic fatty liver disease in humans can be separated into NASH and non-NASH. Of the patients with steatosis alone, up to 20–30% can progress to NASH over 3 years [9], and it appears that the progression into NASH is more likely in the setting of associated risk factors, including insulin resistance, diabetes, and obesity [10, 11]. Various studies show that 20–50% of patients with NASH will display disease progression, either in the form of increased inflammation or liver fibrosis [12–14]. The presence of necrotic-inflammation heralds NASH progression into fibrosis [15]. Patients with NASH can develop HCC even in the absence of fibrosis and cirrhosis, with studies showing a 2–20% 5-year cumulative HCC incidence [16, 17]. Factors leading to progressive NASH and inflammation are not well understood, although it has been agreed upon that multiple ‘pathogenic hits’ are required for the progressive development of liver disease.

Developing a mouse model that closely represents features and progression of NAFLD in human disease has been a challenge. Of the different types of animal models of NAFLD/NASH, none demonstrates the entire spectrum of metabolic derangements and disease progression in humans. For example, there are studies from high fat diet (HFD) feedings that report features of steatohepatitis, fibrosis, and tumor development but lack the earlier steatosis phenotype [18, 19]. The genetically modified models show elements of NAFLD, but thus far have not shown the progression that mimics each stage of human NAFLD. The methionine and/or choline deficient diet models of NASH produce steatohepatitis with significant weight loss that is not characteristic of the human disease [20].

Increasingly, NAFLD progression to NASH with inflammation has been proposed to involve inflammasome activation [21–23]. Inflammasome are multi-protein complexes that play a key role in the modulation of innate immune responses. Inflammasomes function as sensors of endogenous and exogenous damage-associated molecular patterns (DAMPs) and pathogen-associated molecular patterns (PAMPs), respectively which are produced during NAFLD progression to NASH. DAMPs are associated with ROS production, which are known to induce inflammasome activation resulting in the cleavage of effector pro-inflammatory cytokines such as pro-IL-1β and pro-IL-18 [24]. Given the key role played by inflammasome activation in mediating pathogenic hits during hepatic inflammation in NASH, mouse models of progressive NASH leading to tumor formation should characterize this signaling pathway.

In this study we present findings that a high fat diet with high cholesterol and a high sugar supplement (HF–HC–HSD) feeding to mice over 8-, 27- and 49-weeks results in progression of NAFLD to NASH, fibrosis and early liver tumor formation. We report that this diet formulation causes cumulative progressive ‘pathogenic hits’ in well characterized sterile and microbial danger signals resulting in steatosis, inflammation, fibrosis and ultimately tumorigenesis that closely mimic NAFLD progression in humans.

## Methods

### Animal studies

This study was approved by the Institutional Animal Care and Use Committee of the University of Massachusetts Medical School. Principles of laboratory animal care were followed. The experimental time line and setup of mouse feeding on chow diet and our novel high fat, high cholesterol, high sugar diet (HF–HC–HSD) is outlined in Additional file 1: Figure 1S. Male C57Bl/6 wild-type mice that were 8–10 weeks old (n = 6–12 per group) (Jackson Laboratory, Bar Harbor, ME, USA) were fed either a control chow diet or a Western diet equivalent high-fat, high-carbohydrate diet [Surwit diet (58% kcal 35 g% fat)] supplemented with 10% cholesterol (Research Diets, New Brunswick, NJ, USA); with drinking water supplemented with a high-fructose corn syrup equivalent consisting of a total of 42 g/L of carbohydrates at a ratio of 55% fructose (Acros Organics, Morris Plains, NJ, USA) and 45% sucrose (Sigma-Aldrich, St. Louis, MO, USA) by weight, partially based on the study by Kohli et al. [19]. The mice were given ad libitum access to the food and water for 8, 27 or 49 weeks. See Additional File 1: Figure 2S for diet details.

### Biochemical analysis

Serum alanine aminotransferase (ALT) levels were measured using a kinetic method (D-TEK, Bethlehem, PA, USA), and liver triglyceride levels were determined using an L-type triglyceride H kit (Wako Chemicals USA, Inc., Richmond, VA, USA), as we have previously described [25]. ATP in the serum was measured by a chemiluminescence assay (CellTiter-Glo, Promega Corp., Madison, WI, USA). Uric acid in the serum was measured by a fluorometric assay (Abcam, Cambridge, MA, USA). Serum endotoxin was measured using a cell based HEK-blue LPS detection kit (Invivogen, San Diego, CA, USA). Blood glucose was measured using the CVS advanced glucose meter (AgaMatrrix Inc., Salem, NH, USA).

### Caspase activity assay

Caspase-1 activity was determined using freshly prepared whole liver lysates with a colorimetric assay. The analysis was based on the cleavage of the WEHD-pNA (Trp-Glu-His-Asp-*p*-nitroanilide) substrate (R&D Systems, Minneapolis, MN, USA).

### Immunoprecipitation and Western blotting analysis

Equal amounts of proteins from liver lysates were precleared with protein A/G beads (Santa Cruz Biotechnology, Santa Cruz, CA, USA). Beads were removed by centrifugation, and supernatants were incubated and rotated overnight at 4°C with 5 μg anti-HMGB1 (Abcam) or normal rabbit IgG (Santa Cruz Biotechnology, Santa Cruz, CA, USA) as IP negative control. The primary antibody used was 2 μg/mL anti-acetyl lysine antibody (Abcam, Cambridge, MA, USA). Detailed IP protocol was described in a previous manuscript [26]. For western blot analysis of p53, antibodies were obtained from Abcam.

### Cytokine measurements

Mouse IL-1β enzyme-linked immunosorbent assay (ELISA) kit was purchased from R&D Systems (Minneapolis, MN, USA). Mouse MCP-1 ELISA kit was purchased from Biolegend (San Diego, CA, USA).

### Histopathological analysis

Sections of formalin-fixed livers were stained with hematoxylin-eosin and Sirius Red. Frozen samples were also stained with Oil-Red-O that was made at the optimal cutting temperature. Immunohistochemistry staining for cleaved PARP (Abcam, cat. # ab32064), alpha fetoprotein (AFP) (Abcam cat. # ab46799) or sonic hedgehog (shh) (Santa Cruz cat. # sc-9024) was performed in formalin-fixed, paraffin-embedded liver sections according to the manufacturer’s specifications. Slides were analyzed using microscopy using ImageJ and Microsuite (Olympus Soft Imaging Solutions GmbH, Munster, Germany). This histopathology was independently analyzed by a veterinary pathologist (JKP) in a blinded manner (see “Author contributions”).

### Glucose tolerance tests (GTT)

Insulin levels were measured 1 week prior to sacrificing the mice following an overnight fast. Mice were injected i.p. with 40% glucose (Sigma, St. Louis, MO, USA) at 2 mg/kg body weight. Blood was collected by facial vein puncture prior to glucose injection and 30 min after glucose injection. Blood was used for serum insulin measurement using a mouse enzyme-linked immunosorbent assay kit (ALPCO, Salem, NH, USA).

### RNA analysis

RNA was purified using a RNeasy kit (Qiagen Sciences, Germantown, MD, USA) with the on-column DNA digestion (Qiagen Sciences, Germantown, MD, USA). Complementary DNA was transcribed using a reverse-transcription system (Promega Corp., Madison, WI, USA). Real-time quantitative polymerase chain reaction (qPCR) was performed with an iCycler (Bio-Rad Laboratories, Inc., Hercules, CA, USA) using SYBR Green as the detector of double-stranded DNA, as previously described [27].

### Flow cytometry

Isolated liver mononuclear cells (LMNCs) were re-suspended at a concentration of approximately 1 × 10^6^ cells per 50 μL in FACS staining buffer containing anti-mouse CD16/CD32 mAb (2.4G2) from BD Pharmingen (San Jose, CA, USA) to block nonspecific binding to Fcγ receptors and incubated for 10 min at 4°C. Cells were stained with live/dead Fixable Blue Dead Cell Stain Kit from Life Technologies (Grand Island, NY, USA) to gate out dead cells. Further, we added the antibodies: CD11b APC, NK1.1 PE, CD3 Pacific Blue, Ly6C FITC (eBioscience, San Diego, CA, USA) to the cells and incubated for 30 min in the dark at 4°C. For the negative control, the cells were stained with isotype-matched control antibodies (eBioscience, San Diego, CA, USA). The cells were washed with FACS staining buffer by centrifugation at 1,500 rpm for 5 min at 4°C and fixed with 1% paraformaldehyde. The cells were acquired on a BD LSR II instrument (BD Biosciences, San Jose, CA, USA) and the data was analyzed by FlowJo software.

### Electrophoretic mobility shift assay (EMSA)

Electrophoretic mobility shift assay was performed on nuclear proteins extracted from mouse liver samples. Briefly, double-stranded NF-κB oligonucleotide, 5′AGTTGAGGGGACTTTCGC3′ was end labeled with γ^32^P-ATP (PerkinElmer, Waltham, MA, USA) using T4 polynucleotide kinase. Total nuclear protein extract (4 µg) was incubated with 1 µL labeled oligonucleotide (50,000 cpm) and 4 µL dI-dC (Affymetrix Inc., Santa Clara, CA, USA) and 5× gel buffer [containing 20 mM HEPES pH 7.9 (Sigma, St. Louis, MO, USA), 50 mM KCl (Sigma, St. Louis, MO, USA), 0.1 mM EDTA (Boston BioProducts Inc., Ashland, MA, USA), 1 mM DTT (Sigma, St. Louis, MO, USA), 5% glycerol (Fisher Scientific, Fair Lawn, NJ, USA), 200 µg/mL BSA in sterile water] to a final 20 µL final volume made up with nuclease-free water. Cold competition reaction used 20-fold excess NF-κB specific unlabeled double-stranded oligonucleotide to the reaction mixture 20 min prior to adding the ^32^P-labeled oligonucleotide. Reaction samples were incubated at room temperature for 20 min and resolved on a 4% polyacrylamide gel. Gels were dried and exposed to an X-ray film at −80°C for 6 h. Kodak X-OMAT 2000A Processor was used for X-ray film development.

### Statistical analysis

Statistical significance was determined using the nonparametric Kruskal–Wallis test and the Mann–Whitney test when appropriate. Data are presented as means and standard errors and are considered statistically significant for p ≤ 0.05.

## Results

### HF–HC–HSD results in increased liver/body weight ratio and elevated insulin levels

**Figure 1:**
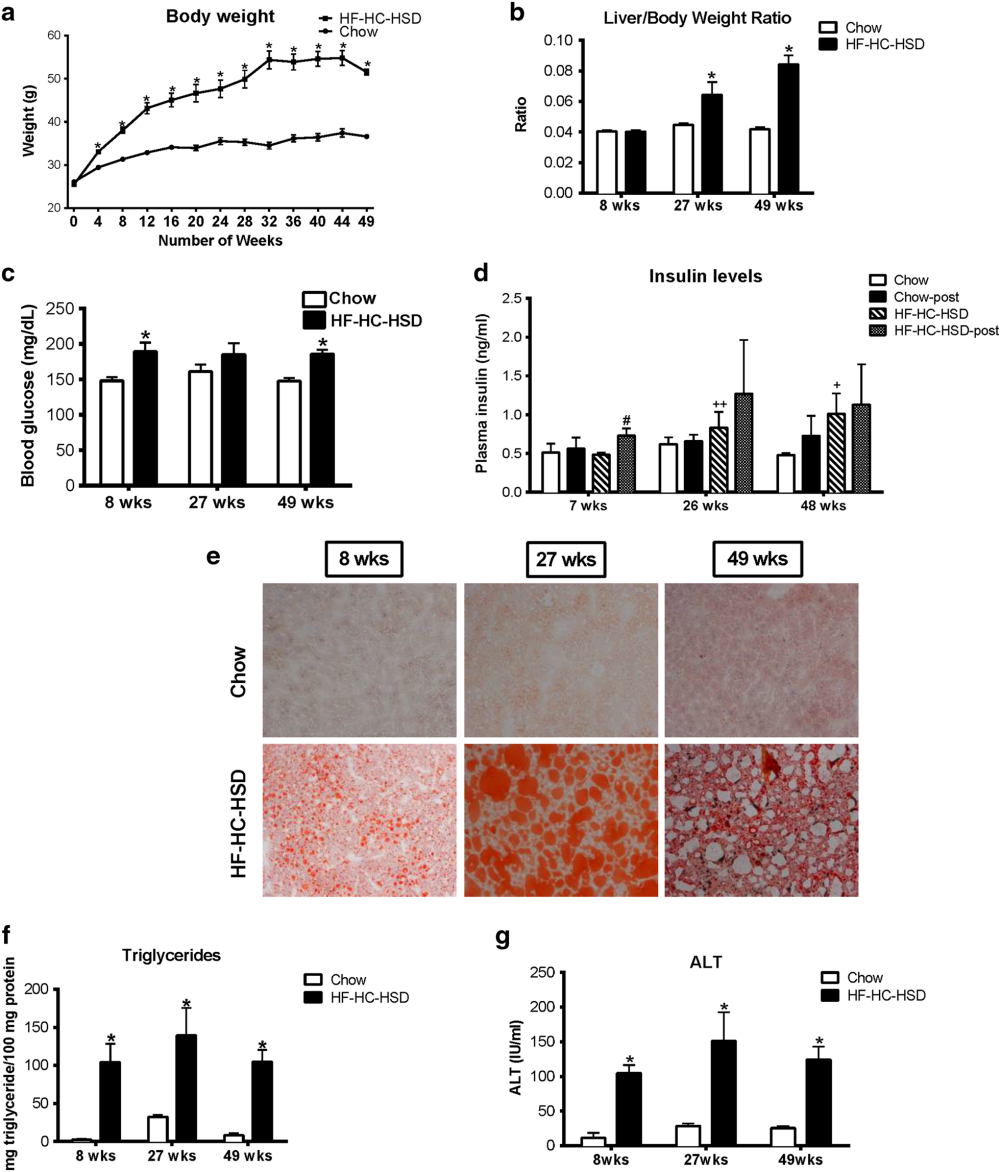
HF–HC–HSD leads to an increase in liver to body weight ratio, insulin resistance and steatohepatitis. Male C57Bl/6 mice were fed with either a high fat diet supplemented with 10% cholesterol and sucrose and fructose in the drinking water, or with a control chow diet for 8, 27, or 49 weeks. Body weight over time (**a**) and liver to body weight ratio (**b**) is shown at each time point. Blood glucose (**c**) over time is shown for each time point. Insulin levels are shown in the chow fed and HFD fed mice both before and 30 min post glucose injection (**d**). Liver tissue was subjected to (**e**) Oil-Red-O staining. One representative slide from n = 6/group is shown. Liver triglyceride levels (**f**) and serum alanine aminotransferase (ALT) levels (**g**) were measured at each time point. *p < 0.01. n = 6–12/group. ^#^p < 0.01—compared to HFD prior to glucose injection, ^+^p < 0.01, ^++^p = 0.02—compared to mice fed chow diet prior to glucose injection. n = 6–12/group.

Mice on a HF–HC–HSD showed sustained weight gain throughout the 49-week feeding period compared to chow diet-fed controls (Figure 1a). We chose to study male mice based on our previous observation that only male and not female mice develop steatohepatitis and insulin resistance after 16 weeks of HFD [21]. In the male mice, weight differences were statistically different at all time points starting at week 4 (Figure 1a). There was no significant difference in the liver/body weight ratio at the 8-week time point, but the liver–body weight ratio was significantly increased at both 27 and 49 weeks in the HF–HC–HFD group compared to mice on the chow diet (Figure 1b).

To test insulin resistance, a characteristic of human NAFLD/NASH, serum insulin levels were evaluated before and 30 min after glucose injection. Baseline serum glucose was higher in HF–HC–HSD fed mice at all time points and significant at weeks 8 and 49 compared to match controls (Figure 1c). Baseline serum insulin levels prior to glucose injection showed a significant increase at 27 and 49 weeks in mice fed a HF–HC–HFD compared to controls (Figure 1d). Serum insulin at 30 min after glucose injection showed an increase at 7, 26, and 48 weeks in mice with HF–HC–HFD compared to chow controls, however the increase was statistically significant only at 8 weeks most likely owed to high individual variability (Figure 1d).

### Steatosis is present throughout the HF–HC–HSD feeding

Obesity and high insulin levels are often associated with fatty liver in humans [10, 11]. In mice that received the HF–HC–HSD, we found liver steatosis as assessed by Oil-Red-O staining (Figure 1e). Liver triglyceride levels were significantly and equally increased in the HFD compared to the chow diet group at all time points (Figure 1f). Liver damage, assessed by serum ALT, was increased at all time points on HF–HC–HSD compared to chow diet (Figure 1g).

### Liver inflammation and inflammatory cytokines gradually increase over time in the HF–HC–HSD feeding

**Figure 2:**
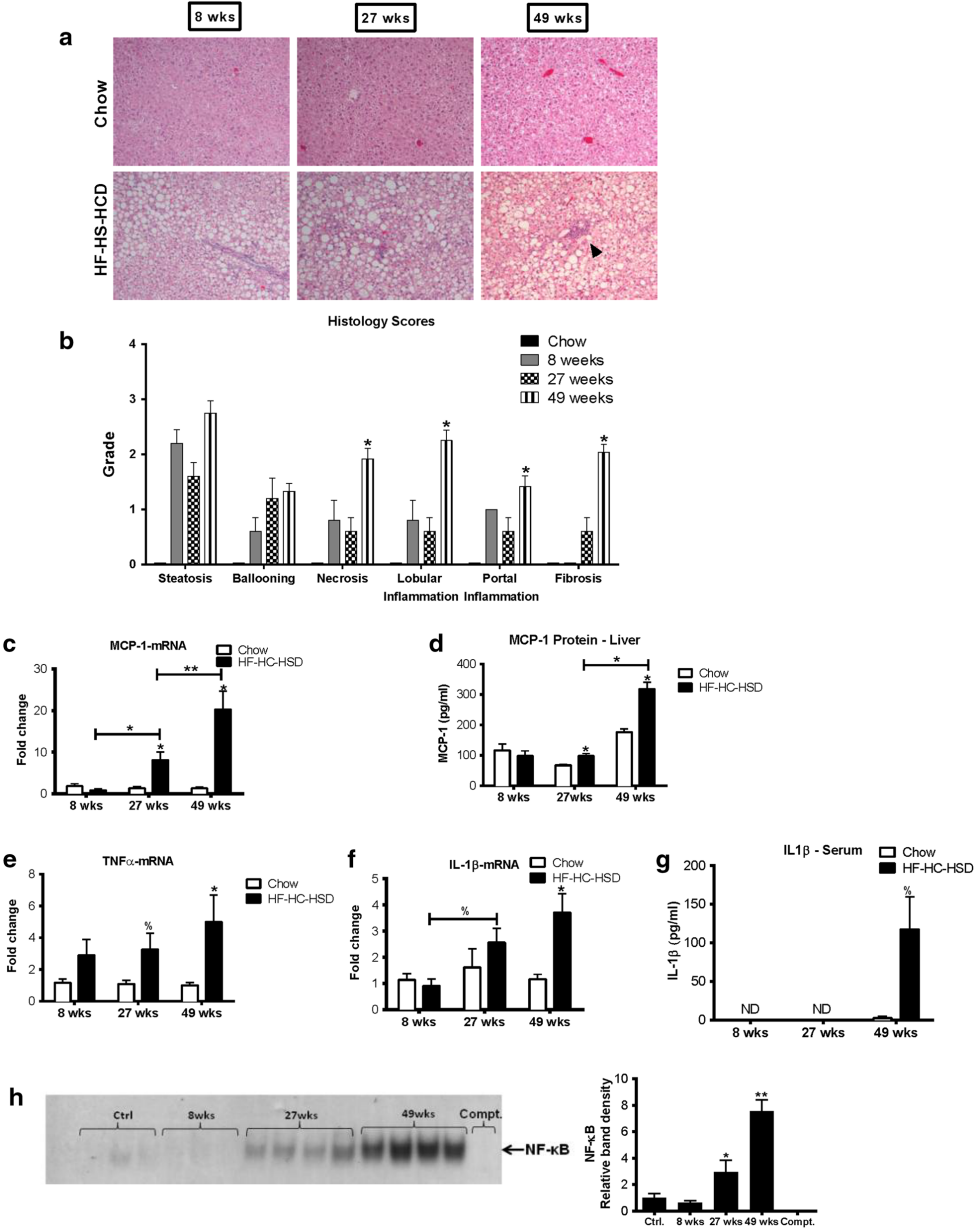
Long-term HF–HC–HSD results in hepatic inflammation. Representative H&E (**a**) slides of chow and HFD fed mice are shown at each time point. Histology scoring was performed on tissue sections at 8, 27 and 49 weeks (**b**). MCP-1 levels in the liver were evaluated at both the **c** mRNA and **d** protein level. Liver TNFα mRNA was evaluated (**e**). We also looked at IL-1β mRNA in the liver (**f**) and IL-1β protein levels in the serum (**g**). IL-1β was undetectable in the serum at 8 and 27 weeks. Electrophoretic mobility assay was performed from nuclear extracts for NFκB at 8,27 and 49 weeks compared to control chow diet (**h**). *p < 0.01, **p = 0.02, ^%^p = 0.05 versus chow fed controls, n = 6–12/group.

Hematoxylin and Eosin (H&E) staining revealed the presence of inflammatory cells in HF–HC–HFD liver (Figure 2a) and histology scoring by a histo-pathologist confirmed the presence of inflammation at 8, 27 and 49 weeks (Figure 2b). The extent of inflammation gradually increased from lobular (8 weeks) to portal (27 weeks) inflammation. Hepatocyte ballooning [sonic hedgehog (shh) positive] [28] (Additional file 1: Figure 3S) increased at 27 weeks and fibrosis was detectable at 27 weeks. Necrosis, lobular, portal inflammation and fibrosis became prevalent at 49 weeks (Figure 2b). This data suggests that robust inflammation in the liver in this mouse model occurs at the later stage of NASH by 49 weeks of HFD feeding.

Next, we evaluated specific markers of inflammation. The chemokine monocyte chemoattractant protein 1 (MCP-1) contributes to steatosis and inflammatory cell infiltration, and is increased during fibrogenesis [29, 30]. There was a significant increase in MCP-1 at both the mRNA (Figure 2c) and protein (Figure 2d) levels at 27 and 49 weeks. Furthermore, the increase in MCP-1 at 49 weeks was significantly higher compared to 27 weeks of feeding with HF–HC–HSD. Tumor necrosis factor α (TNFα) mRNA was significantly increased in the liver at 27 and 49 weeks HF–HC–HSD feeding compared to chow fed controls (Figure 2e). Interleukin-1β (IL-1β) mRNA expression was increased in the liver at 27 and 49 weeks (Figure 2f) and IL-1β protein levels were significantly increased in the serum at 49 weeks (Figure 2g). It is important to note that IL-1β was undetectable in the serum at the earlier time points (8 and 27 weeks) suggesting that IL-1ß and most inflammatory markers show a significant increase at the later stage of non-alcoholic steatohepatitis. Pro-inflammatory cytokine gene expression is regulated by the nuclear factor kB [26]. We found no significant increase in NF-kB activation and DNA binding in livers after 8 weeks of HF–HC–HSD; however, there was a progressive and significant increase in NF-kB nuclear binding at 27 and 49 weeks compared to controls (Figure 2h).

### Inflammasome components are upregulated after 27 and 49 weeks of HF–HC–HSD feeding

**Figure 3:**
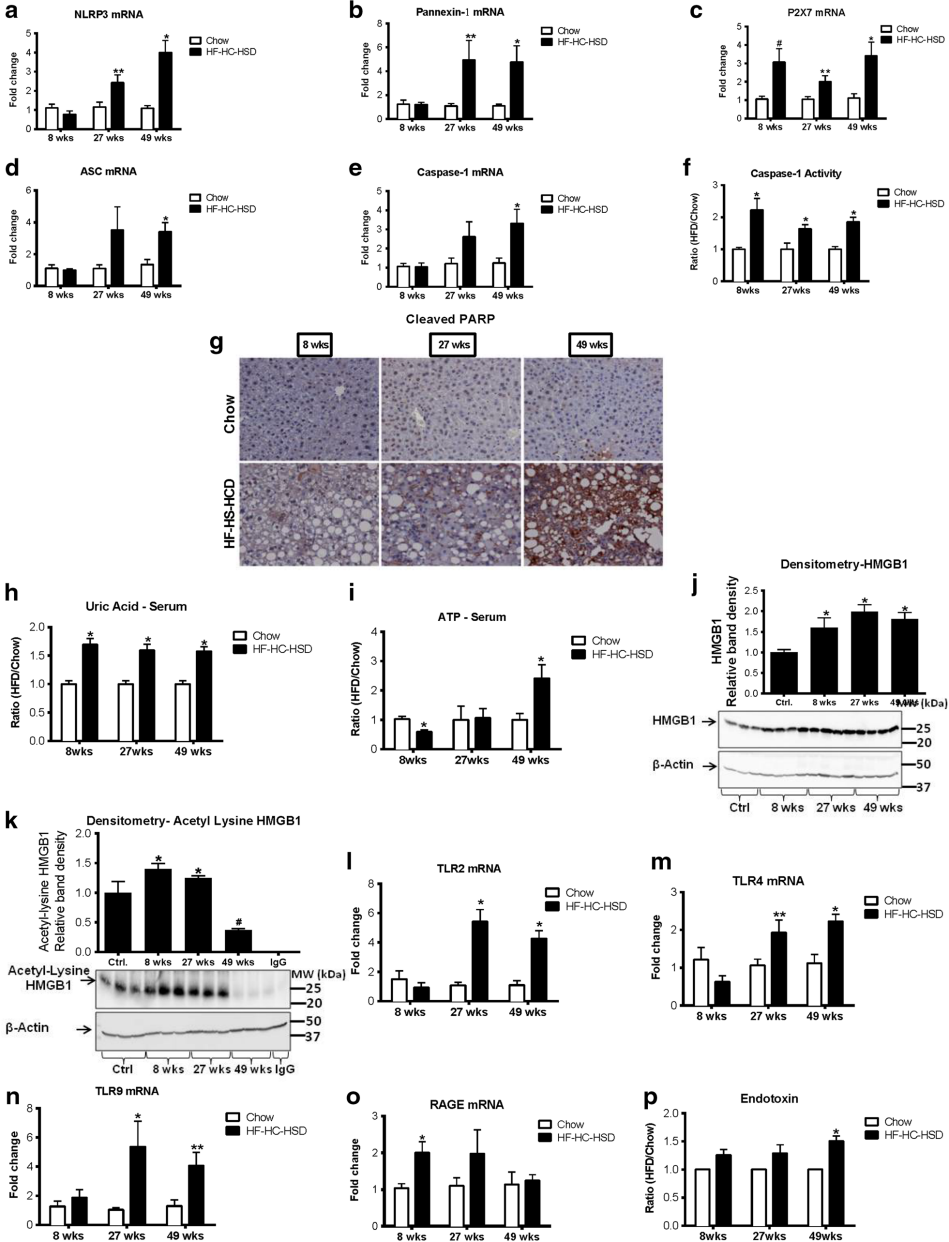
Long-term HF–HC–HSD induces inflammasome upregulation and activation as well as upregulation of danger signals. Hepatic mRNA expression of the inflammasome components NLRP3 (**a**), Pannexin-1 (**b**), P2X7 (**c**), ASC (**d**), and caspase-1 (**e**), were measured using qPCR at each of the different HF–HC–HSD time points. Activation of inflammatory caspases was evaluated by measurement of caspase-1 activity (**f**). Cell death was assessed by immunohistochemistry of cleaved PARP (**g**) on paraffin embedded liver sections. Level of danger signals in the serum, uric acid (**h**), ATP (**i**) were determined. Western blot was performed to determine levels of total HMGB1 (**j**) and immunoprecipitation and western blot for the active form of HMGB1, acetyl-HMGB1 (**k**). Hepatic mRNA expression of receptors for danger signals, TLR2 (**l**), TLR4 (**m**), TLR9 (**n**) and RAGE (**o**) were measured using qPCR. Serum endotoxin was measured at 8, 27 and 49 weeks of HF–HC–HSD (**p**). *p < 0.01, **p = 0.02, ^^^p = 0.03, ^#^p = 0.04 versus chow fed controls, n = 6–12/group.

To explore inflammatory pathways that might be activated in the HF–HC–HSD NASH model, we evaluated the components, ligands and activation of the inflammasome. The inflammasome is a multi-protein complex that includes the NLR sensors and the adapter, ASC, which leads to assembly and activation of pro-caspase-1 leading to its cleavage and activation of caspase-1, which leads to mature IL-1ß production [31, 32]. We found an increase in the mRNA levels of inflammasome sensors, NLRP3 (Figure 3a) and Pannexin-1 (Figure 3b) after both 27 and 49 weeks HF–HC–HSD feeding. P2X7 mRNA was increased at all time points (Figure 3c), whereas ASC (Figure 3d) and caspase-1 (Figure 3e) were significantly increased after 49 weeks HF–HC–HSD. We found increased caspase-1 activity at all time points of the HF–HC–HSD (Figure 3f) which was associated by detectable and high levels cleaved PARP indicating apoptosis at week 49 of HF–HC–HSD feeding (Figure 3g).

### Danger signals accumulate during the progression of NAFLD/NASH in the HF–HC–HSD model

Inflammation and inflammasome activation are responses of the liver immune cells to endogenous and pathogen-derived danger signals [33]. Activation of the inflammasome typically occurs in response to two signals, triggering a TLR and NLR-mediated activation [24, 31–33]. To identify potential triggers of inflammation in the HF–HC–HSD model of NAFLD/NASH, we evaluated the presence of sterile and microbial danger signals that are known to activate the inflammasome. We found a modest, 1.5-fold, increase in serum uric acid levels, an activator of the NLRP3 inflammasome, at all time points in HF–HC–HSD mice compared to controls (Figure 3h). The levels of ATP, a danger signal released by damaged cells that stimulates the inflammasome via the P2X7 receptor, showed a significant increase only at 49 weeks (Figure 3i). High-mobility group box1 (HMGB1) protein is a nuclear protein that is released by stressed and damaged cells into the cytosol [34]. We found an increase in total HMGB1 levels in the liver after feeding with HF–HC–HSD with a maximal increase at 27 weeks (Figure 3j). The activated form of HMGB1, acetyl-HMGB1, was also increased at both 8 and 27 weeks HF–HC–HSD (Figure 3k). We also found an increase in the mRNA levels of the receptors that can sense HMGB1, including TLR2 (Figure 3l), TLR 4 (Figure 3m), TLR9 (Figure 3n) and RAGE (Figure 3o).

Finally, we evaluated the serum levels of the gut-derived exogenous danger signal, endotoxin, (lipopolysaccharide, LPS). LPS is a component of Gram negative bacteria, a major activator of Kupffer cells in NASH, which typically is the first signal in inflammasome activation [35]. Importantly, LPS was significantly increased after 49 weeks of HF–HC–HFD although it showed a trend of increase even at the earlier time points (Figure 3p). Liver expression of the LPS receptor, TLR4, at the mRNA level was also increased in the HF–HC–HSD fed mice at 27 and 49 weeks compared to controls (Figure 3m). These results demonstrated that after 49 weeks of HF–HC–HSD a combination of sterile danger signals are present together with increased circulating levels of LPS that all contribute to inflammasome activation and upregulation of the inflammatory cascade.

### Long-term HF–HC–HSD is associated with Kupffer cell/macrophage activation and changes in M1/M2 macrophage phenotype in the liver

**Figure 4:**
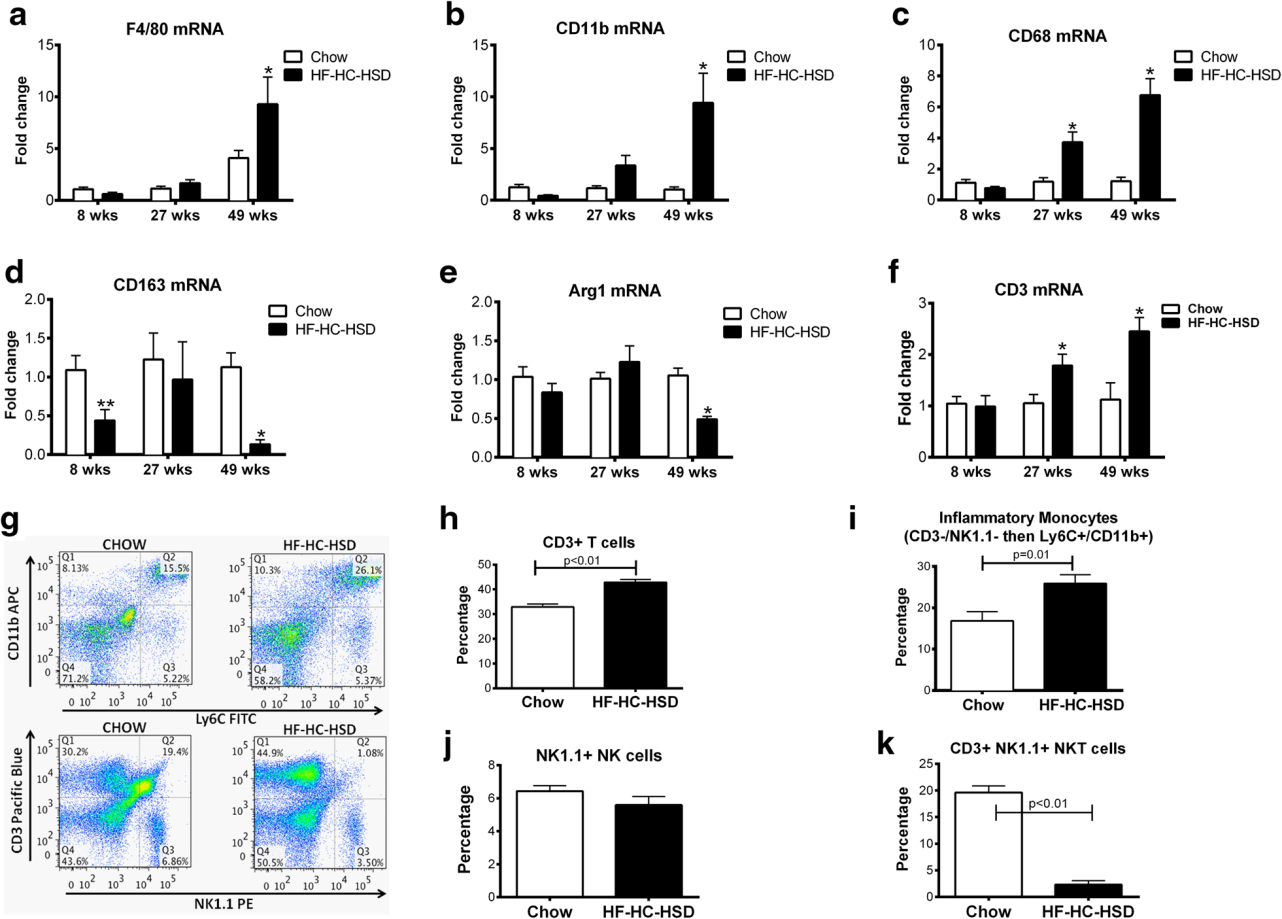
M1 macrophage markers are increased, whereas M2 macrophage markers are decreased with long-term HFD feeding. F4/80 (**a**), CD11b (**b**), and CD68 (**c**) mRNA levels were evaluated using qPCR to look for the presence of M1 macrophages. qPCR was also used to look for the presence of M2 markers CD163 (**d**) and Arg1 (**e**) mRNA levels. mRNA expression of CD3 in liver samples was determined by qPCR (**f**). FACS was performed to determine levels of CD3 (**g**, **h**), inflammatory monocytes (**g**, **i**), NK cells (**g**, **j**) NKT cells (**g**, **k**). *p < 0.01, **p = 0.02, versus chow fed controls, n = 6–12/group.

Recruitment of inflammatory macrophages and Kupffer cell (KC) activation are key elements in the pathogenesis of NASH [36]. We found a progressive increase in the expression of macrophage/KC inflammatory markers F4/80 (Figure 4a), CD11b (Figure 4b) and CD68 (Figure 4c) in the liver with HF–HC–HSD, starting at 27 weeks. In contrast to the progressively increasing expression KC/macrophage markers, M2 macrophage markers, CD163 (Figure 4d) and Arg1 (Figure 4e), were both significantly decreased in the 49-week HF–HC–HSD fibrotic livers suggesting the predominance of M1 macrophages.

CD3 mRNA (Figure 4f) expression increased at 27 weeks on HF–HC–HSD feeding. Flow cytometry analysis of liver mononuclear cell populations after 49 weeks of HF–HC–HSD, revealed an increased CD3+ T cell (Figure 4g, h) and inflammatory monocyte population, identified by CD3−/NK1.1− then CD11b+/Ly6C+ [37] (Figure 4g, i), which is consistent with the increase in inflammatory cells in the long-term high-fat/sugar diet feedings [38–40]. There was however no significant change in NK cell population after 49 weeks of HF–HC–HSD (Figure 4g, j). We observed a reduction in NKT cell population in the liver after 49 weeks of HF–HC–HSD compared to match controls (Figure 4g, k) consistent with the accumulation of inflammatory monocytes as similarly reported [38–40].

Differentiation of monocytes and polarization of macrophages into classical (M1) inflammatory or alternative (M2) macrophages is determined by the tissue environment and contribute to the outcome of inflammation [41]. Consistent with the increase in MCP-1, a macrophage chemoattractant, at 27 weeks (Figure 2c, d), increase in inflammatory macrophages did not begin until 27 weeks (Figure 4a–c). At 49 weeks, these MØ activation markers were significantly increased compared to either the chow diet mice or the 8 and 27-week HF–HC–HSD fed mice. This pattern of macrophage expression was similar to the expression profile of inflammation and inflammasome activation (Figures 2, 4). The increase in TNFα and MCP-1, as shown earlier, in addition to the increase in KC/macrophage markers, is consistent with an increase in the M1 macrophage population [41].

### Long-term HF–HC–HSD results in liver fibrosis and tumor development

**Figure 5:**
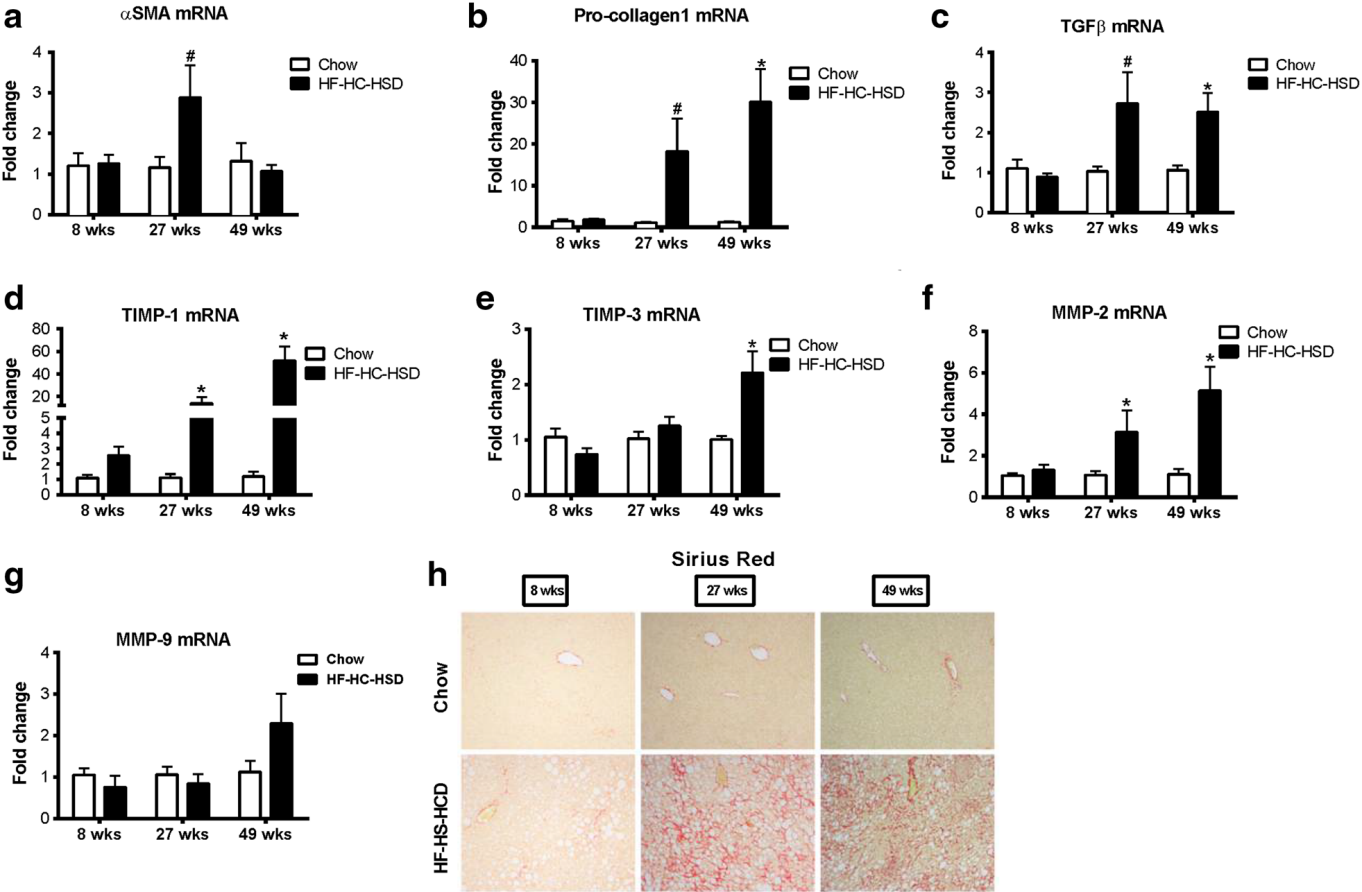
Long-term HF–HC–HSD induces hepatic fibrosis. Hepatic mRNA expression of αSMA (**a**), pro-collagen-1 (**b**), TGFβ (**c**), TIMP-1 (**d**), TIMP-3 (**e**), MMP2 (**f**), and MMP-9 (**g**) were measured using qPCR in male mice at each time point. Representative images of Sirius red staining to evaluate fibrosis at 27 and 49 weeks are shown (**h**).

**Figure 6:**
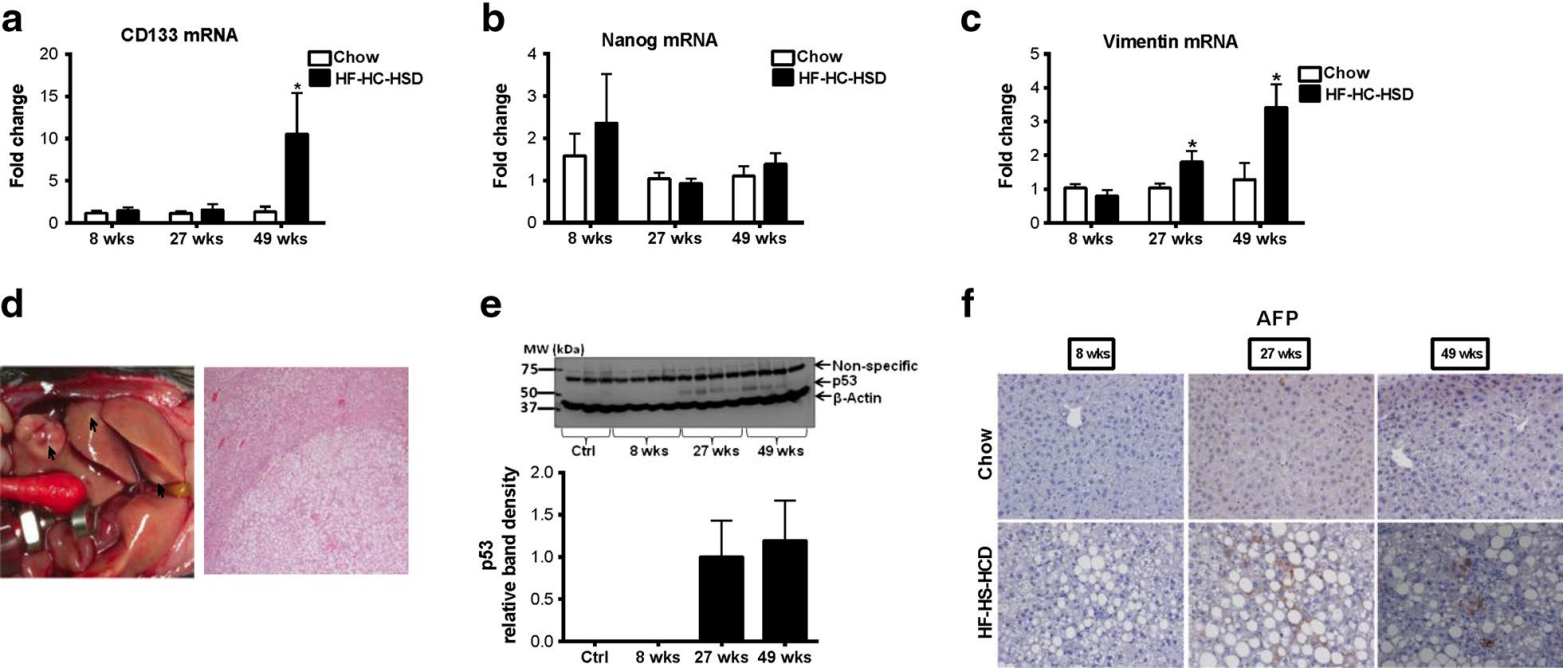
Long-term HF–HC–HSD induces hepatic tumor development. Liver mRNA was determined for CD133 (**a**), Nanog (**b**) and vimentin (**c**) by qPCR. Gross images of tumors that were detected in two of the mice and tumor histopathology (**d**). Detectable liver p53 protein expression was observed by western blotting after 27 and 49 weeks of HF–HC–HSD (**e**). Low but detectable AFP was observed in liver tumor samples after 49 weeks of HF–HC–HSD on immunohistochemistry (**f**). *p < 0.01, ^#^p = 0.03, ^%^p = 0.05 versus chow fed controls, n = 6–12/group.

Inflammation in human NASH is associated with progressive disease and liver fibrosis and predisposes to hepatocellular cancer [42, 43]. We found expression of early markers of fibrogenesis in the liver at 27 weeks and this progressed further after 49-weeks of HF–HC–HSD feeding. Messenger RNA levels of α-smooth muscle actin (αSMA) were significantly increased at 27 weeks (Figure 5a) and pro-collagen1 mRNA was increased at both at 27 and 49 weeks of HF–HC–HSD feeding compared to age matched chow-fed controls (Figure 5b). We also observed an increase in transforming growth factor beta (TGFβ) at both 27- and 49-weeks HF–HC–HSD, consistent with the development of fibrosis at these time points (Figure 5c). Additionally, increased mRNA expression of TIMP-1 (Figure 5d) and TIMP-3 (Figure 5e) was observed from 27- or 49-weeks respectively with significant increase of MMP-2 (Figure 5f) from 27-weeks compared to controls. MMP-9 (Figure 5g) expression levels increased but not significantly at 49-weeks. Sirius red staining indicated fibrosis in livers of HD-HC-HSD mice (Figure 5h). Using histological scoring, there was significantly more fibrosis at 49 weeks than at 27 weeks in the HC–HC–HSD-fed mice (Figure 2b). Additionally, HF–HC–HSD feeding resulted in CD133 (Figure 6a) increase at 49-weeks, no significant change in Nanog (Figure 6b) and a significant increase in vimentin (Figure 6c) from 27-weeks. Finally, after 49 weeks of feeding of HF–HC–HSD we found tumors (or multiple tumors) in 41% (5/12) of the mice; these tumors were classified as hepatic adenomas based on histopathology (Figure 6d). Higher p53 protein levels at weeks 27 and 49 (Figure 6e) and low but detectable AFP (Figure 6f) in some tumor areas though most tumors were negative for AFP. The representative liver tumor H&E histology presented in Figure 6d (HF–HC–HSD 49-weeks) shows cells that closely resemble normal hepatocytes on the upper left, but the neoplastic liver tissue on the central-to-right and lower portion of the presented histology is composed of disorganized hepatocyte cords and does not contain a normal lobular architecture with marked steatosis, but no inflammation, and lacks cytologic atypia consistent with the histopathological observation of a liver adenoma [44, 45]. Additionally, immuno-histology of tumors observed at week 49 shows low but detectable AFP expression suggesting liver progenitor features [46, 47], but most tumor sections at 49 weeks of HF–HC–HSD were AFP negative consistent with liver adenomas [44, 45]. There were no tumors in the chow-fed control group at any time point or after 8 and 27 weeks of HF–HC–HSD.

## Discussion

Non-alcoholic fatty liver disease is an emerging worldwide epidemic yet the understanding of the mechanism and factors that lead to disease progression is limited. Our study describes a NAFLD model in mice that closely mimics the stages of human NAFLD. We show that a close mimic of the Western diet by feeding mice a HFD supplemented with cholesterol and high fructose corn syrup results in progressive development of NAFLD/NASH and fibrosis. While other diet models of NASH use similar fat and glucose diet content, our novel diet has a 10% cholesterol content compared to 0–2% content in previous described diet [48–50]. We found evidence of steatosis without significant inflammation at 8 weeks, consistent with the non-NASH phenotype, in human NAFLD. By 27 weeks, there was evidence of steatohepatitis and early signs of fibrosis and by 49 weeks, NASH progressed to fibrosis and it was associated with liver tumor development. These stages are consistent with the progression of NAFLD in humans [2].

There was a gradual and progressive increase in the inflammatory markers TNF-α and MCP-1 throughout feeding with the HF–HC–HSD, suggesting that inflammation increases as the length of the feeding increases. Both of TNFα and MCP-1 could drive inflammation in NASH. MCP-1 has multiple roles in steatohepatitis. It is a mediator of steatosis and plays a pivotal role in the recruitment of inflammatory cells [29]. MCP-1 administration to primary murine hepatocytes results in triglyceride accumulation [51]. Recruitment of macrophages to the liver was attenuated in MCP-1 knockout mice in alcohol-induced steatohepatitis [52]. Thus, MCP-1 could be one of the driving forces behind steatosis, inflammation, and an increase in liver macrophage population, which we found following a HF–HC–HSD.

IL-1β is an important pro-inflammatory cytokine that amplifies inflammation and sensitizes hepatocytes to TNF induced liver damage [25]. IL-1β secretion is largely the result of the activation of the inflammasome complex [24]. Based on the observation that inflammasome activation began as early as 8 weeks, with an increase in caspase-1 activity, we hypothesize that the inflammasome activation is present with disease progression. The inflammasome has been previously described to play a role in other NASH models [22, 30, 31]. We found a minimal increase in expression of inflammasome components starting at 8 weeks and a more prominent increase was seen at 49 weeks. Furthermore, IL-1β mRNA in the liver and IL-1β protein in the serum were significantly elevated at 49 weeks which was associated with a significant increase in NF-kB nuclear binding. Inflammasome activation requires two signals for IL-1β production [24]. The first signal results in up-regulation of mRNA levels of the inflammasome components, and the second leads to protein production, particularly IL-1β. Our observations suggest that likely danger signals, such as uric acid, ATP, increased NF-kB nuclear binding and LPS are increased in a cumulative way in the HF–HC–HSD feeding and lead to IL-1β production at 49 weeks. Given the significant increase in serum IL-1ß levels at 49 weeks and the equally moderate elevation in caspase-1 activity at 8, 27 and 49 weeks, we cannot rule out that mechanisms other than inflammasome-mediated caspase-1 contribute to the IL-1ß increase in the advanced stage of NASH in our model. Previous studies found pro-IL-1ß cleavage by cathepsin B [53] which may be relevant in our model. This contention is also supported by observations where deficiency of inflammasome components failed to prevent NASH in experimental models [22].

It has been proposed that development of NASH occurs either via multiple hits or parallel hits. The “two-hit model” proposes that after a first hit, in this case steatosis, another hit is needed (potentially LPS) to develop NASH [54]. The multiple parallel hit model was proposed by Tilg and Moschen, who propose that there are multiple hits that act in parallel that lead to NASH, particularly adipose-tissue and gut-derived factors, as well as endoplasmic reticulum (ER) stress [55]. Our model appears to be consistent with the multiple parallel hit models, as it takes consecutive danger signals over time, such as uric acid and HMGB1 initially with subsequent increases in endotoxin and ATP later. The release of endogenous danger signals is highly plausible as necrosis on histology appeared before significant increase in inflammation. We hypothesize that multiple sterile danger signals and cumulative stimulating effects in inflammatory cells are needed for NASH progression.

Damage-associated molecular patterns are known to play a role in liver injury and inflammation [34]. We evaluated the levels of various DAMPs in our HF–HC–HSD feeding and their associated receptors and found an increase in a number of DAMPs. We found that total high mobility group box1 (HMGB1) protein and its active form, acetyl-HMGB1 were elevated, however did not appear to be sufficient to result in inflammation and fibrosis at 8 weeks. We also detected increases in the mRNA levels of the HMGB1 sensing receptors TLR2, TLR4, and TLR9 at 27 and 49-weeks, and an increase in RAGE at 8 weeks. HMGB proteins bind to nucleic acids and are thought to act in complex with other proinflammatory mediators, such as endotoxin and IL-1β, to trigger inflammation [34]. HMGB1 protein levels were also shown to be increased in the livers of mouse fed a methionine–choline deficient diet [23]. In addition to HMGB1, the levels of uric acid, a trigger of Nlrp3 inflammasome activation, are also elevated in the HF–HC–HSD model. Furthermore, an elevation of endotoxin and ATP was only seen after 49 weeks of HF–HC–HSD, and their associated receptors TLR4 and P2X7, respectively, were also increased at this time point. In our model, we see the most significant inflammation after 49 weeks of HF–HC–HSD, where there is an increase in both endotoxin and IL-1β, which may the necessary mediators for NASH progression.

These observations support that the gradual progression of NASH/fibrosis in the HF–HC–HSD feeding is associated with a progressive buildup of danger signals that engage multiple receptors involved in activation of inflammatory cells and pro-inflammatory cytokine pathways with the prolonged metabolic insult of HF–HS–HSD. Consistent with this finding, we found a significant increase in inflammatory cell recruitment in the liver after 27 and 49 weeks of HF–HC–HSD. Macrophages are highly plastic and in the inflamed tissues acquire an activated M1 phenotype. The M2, alternatively activated macrophages express CD163, Arg1, CD206, and produce IL-10 and TGFβ with an overall anti-inflammatory function. In a previous study, an increase in M1 macrophages in the liver after a 27 week HFD feeding was seen [56]. This group also found an increase in M2 marker expression in these mice as well as in patients who were morbidly obese. In our study, we observed an increase in M1 macrophages at the later time points of the HF–HC–HSD; however in contrast to Wan et al. [56], we found a decrease in expression of M2 macrophage markers (CD163, Arg1). The differences that are seen in the M2 population could be due to the different diets used as well as the fact that the mice were fed for 49 weeks in our experiment, as opposed to 27 weeks in Wan’s study. These changes in the macrophage populations are also likely contributing to the development of inflammation and fibrosis.

We observed a gradual increase in pro-collagen1, TGF-β, TIMP-1, TIPM3 and MMP2 markers for fibrosis [43, 57], whereas αSMA peaked at 27 weeks and decreased at 49 weeks, this pattern of fibrosis markers was consistent with previous findings that αSMA is elevated earlier in the development of fibrosis [57, 58]. Progression of fibrosis was also evident on Sirius red staining and histology analysis. Following fibrosis our HF–HC–HSD induced detectable p53 in the liver by weeks 27 and 49 as well as tumor development by week 49.

## Conclusions

In summary, the HF–HC–HSD feeding in mice closely mimics the progression of human NASH. In this model of NASH, we identified a gradual increase in both endogenous (HMGB1, uric acid, ATP) and exogenous (LPS) danger signals along with up-regulation of their respective receptor sensors, supporting that multiple cumulative danger signals from metabolic insult results in the progression of NAFLD to NASH, fibrosis and liver tumors. While our data describes some mechanistic signaling modulations associated with HF–HC–HSD induced liver neoplasia, they are by no means an exhaustive list.

## Additional files

Additional file 1:
**Figure 1S: Experimental outline for mice on chow or HF-HC-HSD.** 8–10 week old C57BL6 mice were fed with chow diet or HF-HC-HSD for the indicated time points and euthanized as indicated. All mice received unrestricted access to food and water during the entire experiment. Mice bedding and cages were changed every 2 days for the entire feeding period. Additional file 2:
**Figure 2S: Diet details of HF-HC-HSD.** The composition of the high fat-cholesterol-sugar diet shows the detailed ingredient contents (gm % and Kcal %) of protein, carbohydrate, fat, casein, DL-Methionine, maltodextrin, sucrose, soybean oil, hydrogenated coconut oil, sodium bicarbonate, potassium citrate, vitamin mix, choline Bitartrate, cholesterol and dye present. Additional file 3:
**Figure 3S: Liver immunohistology after 49 weeks HF-HC-HSD.** Ballooned hepatocyte showing positive sonic hedgehog staining.

## Abbreviations

NAFLD: non-alcoholic fatty liver disease
HF–HC–HSD: high fat diet with high cholesterol and a high sugar supplement diet
NASH: non-alcoholic steatohepatitis
HFD: high fat diet
MCP-1: monocyte chemoattractant protein 1
TNFα: tumor necrosis factor α
IL-1β: interleukin-1β
αSMA: α-smooth muscle actin
TGFβ: transforming growth factor beta
HMGB1: high-mobility group box1
