# Supplementary figures and images for: Progression of non-alcoholic steatosis to steatohepatitis and fibrosis parallels cumulative accumulation of danger signals that promote inflammation and liver tumors in a high fat–cholesterol–sugar diet model in mice
Source: J Transl Med. 2015 Jun 16;13:193. doi: 10.1186/s12967-015-0552-7 (PMC4467677; doi:10.1186/s12967-015-0552-7)

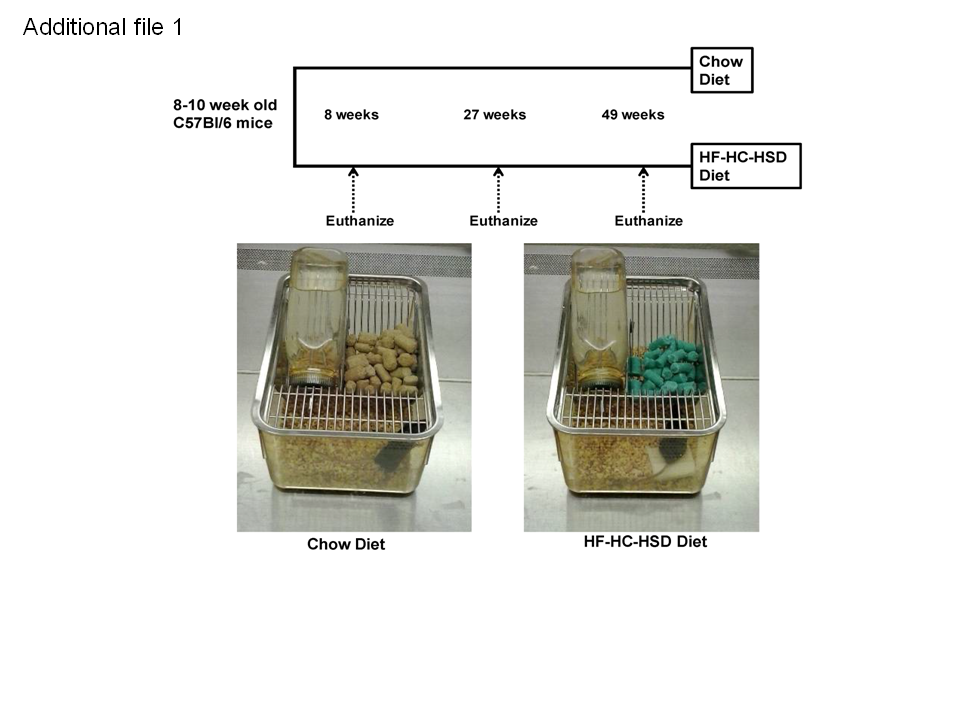

Supplement: Additional file 1: — Figure 1S: Experimental outline for mice on chow or HF-HC-HSD. 8–10 week old C57BL6 mice were fed with chow diet or HF-HC-HSD for the indicated time points and euthanized as indicated. All mice received unrestricted access to food and water during the entire experiment. Mice bedding and cages were changed every 2 days for the entire feeding period. [file 12967_2015_552_MOESM1_ESM.tiff]

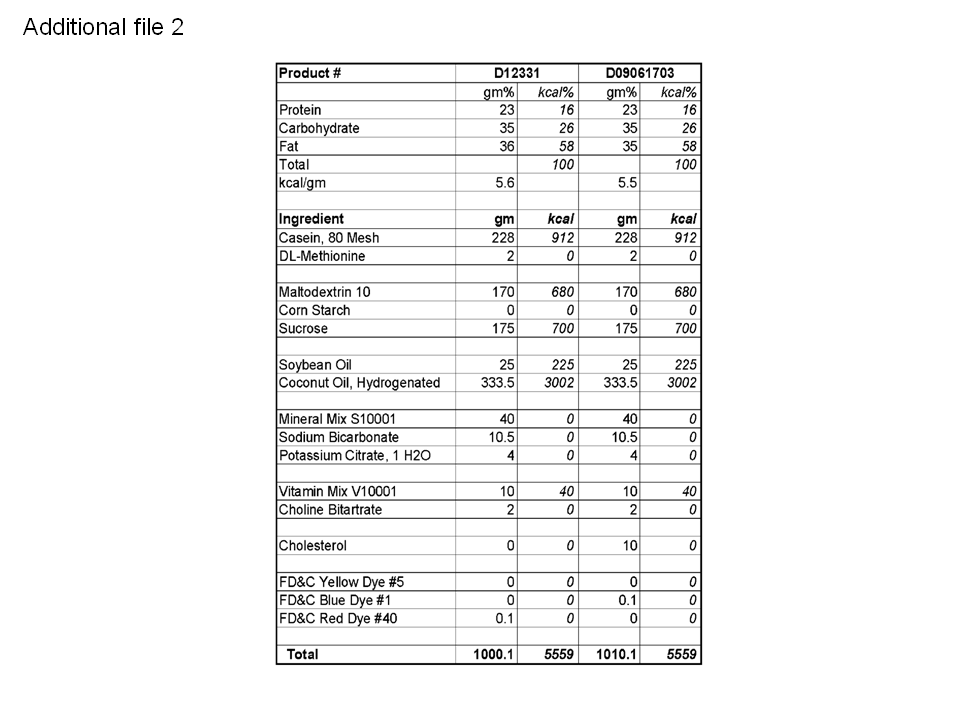

Supplement: Additional file 2: — Figure 2S: Diet details of HF-HC-HSD. The composition of the high fat-cholesterol-sugar diet shows the detailed ingredient contents (gm % and Kcal %) of protein, carbohydrate, fat, casein, DL-Methionine, maltodextrin, sucrose, soybean oil, hydrogenated coconut oil, sodium bicarbonate, potassium citrate, vitamin mix, choline Bitartrate, cholesterol and dye present. [file 12967_2015_552_MOESM2_ESM.tiff]

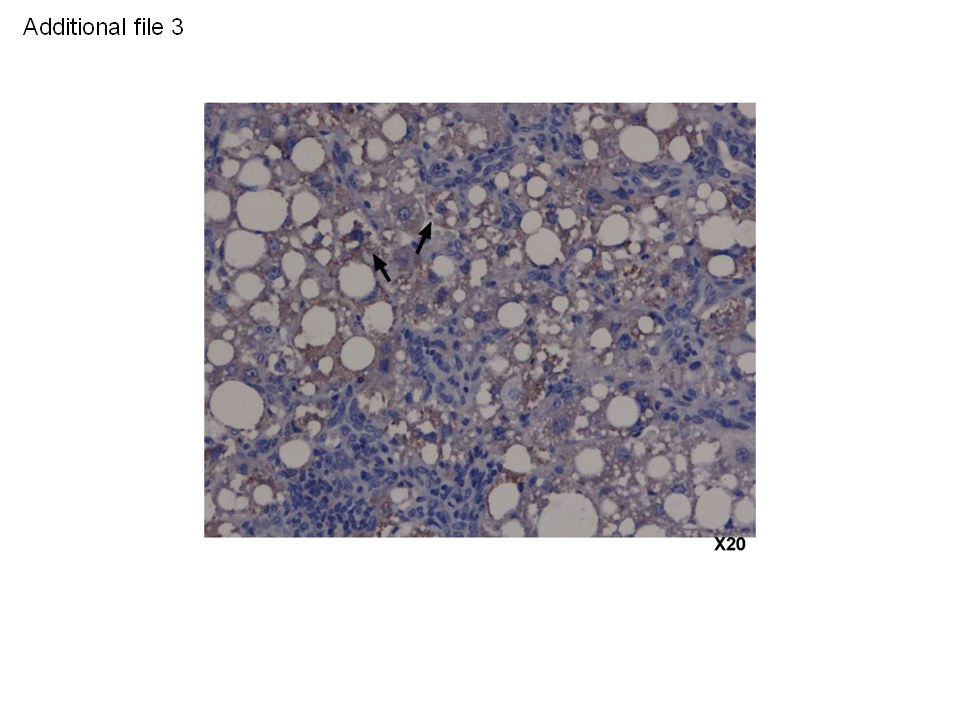

Supplement: Additional file 3: — Figure 3S: Liver immunohistology after 49 weeks HF-HC-HSD. Ballooned hepatocyte showing positive sonic hedgehog staining. [file 12967_2015_552_MOESM3_ESM.tiff]
